# Plea Bargains as Drivers of Incarceration-Related Health Outcomes

**DOI:** 10.1017/jme.2025.58

**Published:** 2025

**Authors:** Riley Smith

**Affiliations:** Boston University School of Public Health

**Keywords:** plea bargaining, incarceration, public health, anti-racism, policy analysis

## Abstract

The discipline of public health has begun to recognize the structural inequities of the carceral system as drivers of poor individual and population health. The number of people incarcerated and the length of their incarceration determine the scope and gravity of their exposure to these individual and public health effects. Plea bargains all but guarantee a period of incarceration, often for many years, because prosecutors have significant bargaining power against defendants who often do not fully understand their rights or the likelihood of receiving the sentences that prosecutors would be seeking in trial. I propose and analyze several pathways through which to eliminate or severely restrict the practice of plea bargaining to minimize the health effects associated with incarceration. I conclude that state legislation would be most feasible and effective at eliminating plea bargains but, without concurrent interventions addressing mandatory minima and/or bail, would not fundamentally address the primary concerns of sentence length and overcrowding.

## Incarceration as a Public Health Issue

Public health research and principles have evolved an ever-broadening understanding of the forces affecting health. Public health experts have adopted the Social Determinants of Health framework to identify the multifaceted and interconnected ways that our systems, institutions, communities, and selves affect health at every level. From this perspective, the discipline of public health has begun to recognize the structural inequities of the carceral system as drivers of poor individual and population health, which upstream and downstream interventions can target.

In the US,^1^ 2 million people (565 per 100,000 residents) are in some way confined by the State, either through imprisonment, probation/parole, home confinement, or pre-trial detention.^2^ The Eighth Amendment to the Constitution requires that incarcerated people receive some minimum standard of necessary care during periods of State confinement.^3^ However, currently and formerly incarcerated people and their allies, as well as many healthcare providers and health experts, frequently highlight the ways in which jails and prisons cause and exacerbate physical and mental illness and disease.

### Disease Transmission

Prisons and jails are prime sites for rapid and unmanageable disease transmission both within the walls and out in the communities that prison staff return to when their shift ends. These facilities are overpopulated and poorly ventilated, and part of the punitive process includes extremely restricted and regimented movement. Prisoners do not have the autonomy to isolate themselves from others, nor do they have access to personal protective equipment (PPE) or other preventative measures to minimize transmission risk of infectious diseases. Staff bring in any contagions they may have which can then rapidly spread within the facilities. Similarly, staff who become infected with communicable diseases bring these diseases back to their communities. During the COVID-19 pandemic, prisons were a primary site of mass infection, and many advocates argued for depopulating the prisons, especially of those who were particularly vulnerable to COVID infection, as a means of slowing the spread of the disease.

### Sanitation and Hygiene

The COVID-19 pandemic also highlighted the ways in which prison procedures fail to provide adequate sanitary conditions within their facilities. Prisoners often must purchase sanitizers, cleaning supplies, soaps, body washes, and menstrual products through the commissary. These items must be purchased by money either earned through work — where wages can be pennies an hour in parts of the country — or money donated by family and friends in the free world.^4^ This shifting of sanitary responsibility onto prisoners, of whom most are unable to afford necessary products to meet minimum sanitation standards, fosters an environment of poor personal hygiene and environmental sanitation.

### Medical Care

Despite being constitutionally entitled to medical care, prisoners and many medical staff report inadequate or wholly absent care for everything ranging from small injuries to chronic illnesses. Prisoners report misdiagnoses, unnecessary invasive procedures, poor maternal healthcare, and more. Many are inhibited from seeking care in the first place, as they are only allowed to receive medical treatment if a correctional officer brings them to the medical wing.^5^ Necessary care can be delayed indefinitely with little recourse as reports of medical neglect are largely ignored.

### Trauma

Incarceration is a traumatic experience. Incarcerated individuals are caged, dehumanized, violated, punished, and otherwise mistreated, and this is justified by the societal agreement that these individuals deserve whatever happens by virtue of being a criminal. Additionally, many individuals who enter these facilities — including 90% of women — have experienced traumatic events prior to interacting with the carceral system, and the conditions of their confinement prevent them from recovering and healing while further traumatizing them.^6^

These effects and others are felt long after a person is released from prison as well. Though in theory a person’s punishment ends when their sentence does, the effects of the punishment often continue indefinitely. For example, criminal records often disqualify people from employment and housing^7^ with minimal recourse. Further, the stigma associated with incarceration can be incredibly isolating, preventing formerly incarcerated people from getting basic needs met.^8^ Additionally, incarceration impacts the families and communities of those incarcerated because of the emotional, financial, and social challenges caused by their absence. It is estimated that 80 million individuals in the United States have a criminal record and nearly half of all adults in the United States have immediate family members who are currently or formerly incarcerated.^9^

The burden of these poor health and community outcomes is disproportionately borne by people of color, especially Black people. Black people are overrepresented in prison populations due to policies that increase police presence in predominantly Black communities, laws targeting behaviors associated with communities of color, and discriminatory actions taken at all levels of the legal process, from arrest to conviction to release.^10^ These practices were introduced to reinforce white power as slavery was being abolished, systematizing racial disparities as fundamental to the operation of the modern carceral system.^11^ Black and brown communities experience higher rates of disease prevalence, especially infectious diseases and those that affect the immune system, which are exacerbated by periods of incarceration.^12^ They are more likely to be arrested, more likely to receive a conviction, and more likely to receive longer sentences for the same crimes as their white counterparts, increasing the likelihood and duration of exposure to the health consequences of incarceration.^13^ These compound other disease burdens, leading to more significant detrimental effects.

The number of people incarcerated, and the length of their incarceration, determines the scope and gravity of their exposure to these individual and public health effects. Therefore, one way to minimize the public health effects of incarceration is to address factors that increase the likelihood of incarceration and lengthy sentences. In this paper, I argue that plea bargains are a driver of incarceration and its subsequent health effects. I propose several pathways through which to eliminate or severely restrict the practice of plea bargaining to minimize the health effects associated with incarceration. Each intervention would be implemented through different government channels and may affect some or all individuals charged with a crime. I determine effectiveness by considering how broad the reduction in the practices is as well as how many people are likely to experience shorter or no periods of incarceration. I determine feasibility through resources, capacity, and political will.

## Plea Bargaining and Its Relationship to Incarceration Length

In the United States, only 2% of all criminal cases ever go to trial, with the rest resulting in plea bargains.^14^ Plea bargains are meant to be voluntary negotiations between prosecutors and defendants that lessen the severity of criminal punishment for defendants for choosing to plead guilty rather than go to trial. Plea bargains are used primarily to prevent system overwhelm by minimizing the number of trials and associated resources to conduct said trials, as made explicit in the Court’s opinion in *Santobello v. New York* where plea bargains were determined to be “an essential component of the administration of justice … to be encouraged.”^15^

The negative health effects of incarceration are exacerbated by time and overcrowding, both of which are increased through plea bargaining. If all individuals charged with a crime went to trial, they would have a fair opportunity to argue their defense and potentially be found innocent of some or all charges. Even if they are found guilty of some charges, they would have the opportunity to argue mitigating circumstances for consideration in sentencing. However, plea bargains all but guarantee a period of incarceration, often for many years.

### Power Imbalances in Plea Bargaining

Plea bargains are a presumed step in the criminal conviction process, occurring imminently after arrest. Prosecutors present all the charges for which they have probable cause to bring to trial, then offer to drop or lower charges in exchange for a guilty plea.^16^ Because prosecutors, not police or judges, determine which charges would ultimately go to trial, they have significant power over the defendant and their counsel.^17^ Prosecutors may present any charge regardless of the strength of their evidence, and need only provide the defense with exculpatory evidence.^18^ Due to the expediency of the court system and the often-limited resources available to defense attorneys, the defense often engages in the process before doing any investigation of their own that may strengthen the defendant’s case.^19^

Defendants have a constitutional right to effective counsel in criminal proceedings.^20^ However, the definition of effective counsel and its application in the plea process places a significant burden on the defendant, who may not know whether the counsel they are receiving meets the parameters. Unlike in a courtroom where all actions are a matter of public record, plea-bargaining occurs largely without oversight.^21^ Defendants who are facing charges with extreme consequences, and who do not know the strength of their defense or the weakness of the prosecution, can be easily swayed to agree to plea deals, especially if they are encouraged by their defense attorney.^22^ These power imbalances — between prosecution and defense and between defense attorney and defendant — create conditions ripe for exploitation, all within legal parameters.

### Racial Discrepancies in Plea Bargaining

While the topic demands further study, emerging research shows stark racial disparities in the types of pleas offered to and accepted by defendants.^23^ Where the defendant has no prior criminal history, Black defendants are more likely than white defendants to receive plea offers that include some period of incarceration, and they are less likely to have charges dropped or reduced.^24^ Additionally, Black defendants are far more likely to accept plea deals that include some period of incarceration compared to white defendants^25^ and are more likely to agree to false pleas.^26^ Racial discrepancies are highest for defendants with so-called “low-level offenses”, which are the vast majority of cases in the carceral system.^27^

Researchers argue that prosecutors use race as a proxy for criminality when determining which charges to file and which to negotiate away. In his 2018 publication, Criminalizing Race: Racial Disparities in Plea Bargaining, Carlos Berdejo supports this conclusion by demonstrating that racial disparities only exist among defendants who have no criminal history and/or are charged with lower-level offenses.^28^ Berdejo argues that, lacking criminal history or severity of criminal behavior as markers for potential dangerousness and future criminality, prosecutors instead rely implicitly or explicitly on race.^29^ Defense attorneys may also use race as a proxy when determining whether to fight for a better deal or pursue trial, although researchers have come to conflicting conclusions on this front.^30^

### False Pleas

Though the literature is limited, those who have conducted analyses of the reasons why those who did not commit a crime plead guilty have found similar themes. The first is concern about the competency of the defense attorney. Defendants who think their defense attorney is unable to represent them effectively in the court room, due to factors such as a feeling that the defense attorney presumes them to be guilty or demonstrated or perceived inability of the defense attorney to put in the appropriate time and resources to build a case, are more likely to take a plea where they know the outcome instead of putting their freedom at even greater risk at trial.^31^ The second is pressure from the defense attorney. Even though the decision to accept a plea or go to trial ultimately rests on the defendant, defense attorneys wield enormous influence over their defendants because of the presumption of expertise — if the attorney who understands the court system better than the defendant is presenting the deal as a good option, the defendant is likely to agree.^32^ Notably, this influence is far less significant on those who committed the crime for which they are charged.^33^ Defense attorneys often approach plea bargains as a calculation of the “plea discount” — the difference in outcomes for a plea deal versus the maximum punishment at trial.^34^ If the discount is high, they are far more likely to advise a defendant to accept a deal regardless of innocence or strength of the defense.^35^ Innocent defendants who are encouraged to go to trial are significantly less likely to choose to plead guilty over those who are encouraged to accept a plea deal, demonstrating the strength of this influence on the defendant’s ultimate decision.^36^

Police officers want to demonstrate that they are effective through high arrest rates, but prosecutors want to demonstrate that they are effective through winning cases. In a system where prosecutors can rely on plea bargaining, the severity of the charges or strength of the evidence is not as important, as the prosecutor is unlikely to have to argue the case before a judge.^37^ Given that 17% of people who go to trial are acquitted^38^ and that nearly a quarter of people who have been exonerated had been incarcerated through a guilty plea,^39^ it is very likely that if all cases went to trial, fewer people would be convicted of crimes and sentenced to incarceration, thus decreasing the incarceration rate in federal and state prisons. In fact, plea bargaining bans have been attempted before, and some argue that these bans have been effective at minimizing the incarceration of individuals who did not commit the crimes they were charged with.^40^ When plea bargaining is not an option, prosecutors have had to be more thorough in their screening processes before determining whether to consider pursuing cases, meaning any charges filed were for cases with a strong amount of evidence.^41^

Incarceration is the greatest infringement on personal freedom and as already discussed has significant negative health effects, especially for Black and brown people. Thus, the decision to incarcerate someone and the process by which that occurs should be held to the highest scrutiny and severely restricted. Plea-bargains necessarily circumvent this level of scrutiny and restriction due to their lack of oversight and the necessity of waiving constitutional rights in order to participate.

Early courts found plea bargaining objectionable.^42^ However, the volume of cases and the presumed voluntary nature of plea bargains has allowed the practice to continue with few restrictions and minimal oversight. Were plea bargains eliminated, state and federal courts would have to try all cases, granting all people charged with a crime the right to argue their innocence or present mitigating circumstances for consideration.

## Potential Interventions to Reduce Exposure to Incarceration via Plea Bargains

In the United States, individuals can be tried for state or federal crimes, and their experience of the carceral system is based largely on the jurisdiction they are tried in. Local, state, and federal laws may affect what is considered a crime and an acceptable punishment. Interventions thus may occur at different levels and branches of government and will affect only those within those jurisdictions. In the following sections, I explore different governmental channels through which actors may severely limit or fully eliminate the practice of plea-bargaining.

### Challenge to Constitutionality

The Constitution supersedes all other governing decisions in the United States. Constitutional questions are heard by federal courts and the Supreme Court, and their interpretations of the Constitution create the benchmark for interpreting future legislation. State and federal governments must defer to the decisions of the judiciary. Thus, federal court interventions would immediately halt these processes until and unless they were overturned.

Rule 11 of the Federal Standards of Criminal Procedure provides the conditions that must be met for a court to accept a guilty plea, including, in part, the court’s determination that a plea was made voluntarily and with a full understanding of the consequences of accepting the plea.^43^ Plea bargains, however, occur outside of the court between the prosecutor and defendant. Since the prosecutor determines which charges will be argued before the judge, they have significant bargaining power. Thus, many legal scholars and advocates argue that guilty pleas made because of plea bargains can never be truly voluntary.^44^

The Court has heard numerous challenges to the constitutionality of plea bargains. While many of these challenges have led to decisions that refined the circumstances under which a plea can be accepted,^45^ the Supreme Court has maintained their fundamental constitutionality. The Court maintains that individuals have the right to waive their constitutional rights “knowingly and voluntarily”, and Fifth and Sixth Amendment rights are no exception.^46^

Perhaps the most significant example of the Court’s failure to address the power imbalance between a defendant and the state is in *Brady v. United States*, a 1970 case that affirmed that even the threat of the death penalty is not significant enough to consider a plea bargain to be compelled or coerced.^47^ In fact, the Court argued that plea bargains are mutually beneficial, and the “advantages of pleading guilty … are obvious” for defendants who “see slight possibility of acquittal.”^48^ *Brady* has been cited in numerous cases upholding the constitutionality of plea bargains and other waivers of rights even in situations of severe power imbalances.

These arguments rest on the presumption that all, or even most, defendants who plead guilty *are* guilty and are merely trying to avoid harsher penalties. While there are indeed many who are seeking this outcome, and while Rule 11 attempts to mitigate egregious abuses,^49^ the structural inequities embedded in the United States carceral system lead to many innocent individuals with minimal knowledge of their rights and/or few resources to fight for their innocence facing criminal charges.^50^ The Court’s continued ignorance of this fact perpetuates a system whereby people who fear they have everything to lose are convinced to sign away their rights for the chance at a slightly more manageable confinement regardless of the facts of their case or the long-term consequences of this choice.

Given how embedded this idea is into the structures of the carceral and judicial systems, a decision by a federal court or the Supreme Court deeming plea bargains unconstitutional would have the most immediate,^51^ broad-reaching downstream impacts, as state and federal courts could no longer accept pleas that are part of a plea bargain. In addition to an immediate stop to all plea bargains, there would be the opportunity for individuals who accepted plea bargains to ask the court for a retroactive application of the ruling, which, if effective, could lead to the release or retrial of numerous individuals interested in challenging their confinement based on the ruling.^52^

Constitutional challenges begin with federal courts across the country. A federal judge could rule plea bargains unconstitutional, and if it was not appealed or the Supreme Court affirmed the decision, that would become the benchmark. Alternatively, the Supreme Court could overturn a lower court’s decision that had affirmed the constitutionality of plea bargains. This process is incredibly time consuming, resource intensive, and uncertain. Challenges have to happen in the right jurisdiction at the right time, and simultaneous challenges in different jurisdictions can lead to conflicting rulings increasing confusion and uncertainty.^53^ Additionally, courts tend to rule under the principle of *stare decisis*, or ruling based on precedent.^54^ The Supreme Court has set and maintained a decades-long precedent affirming the constitutionality of plea bargains.^55^ An overruling of that precedent is incredibly unlikely.

### Federal Legislation

The Constitution restricts the power of the federal government to legislate over the United States. Congress is limited to those powers explicitly laid out in the constitution, such as the power to tax, spend, and regulate interstate commerce. Through the Necessary and Proper Clause, Congress has the power to “make rules governing the practice and pleading in [lower federal] courts.”^56^

In 1934, Congress passed the Rules Enabling Act which allowed the Supreme Court to propose procedural rules and amendments but still grants Congress final authority on whether these proposals are enacted.^57^ One set of rules, the Federal Rules of Criminal Procedure, governs all federal court proceedings, including Rule 11, which dictates procedural and conditional expectations surrounding pleas and plea bargains.

While Congress abdicated “primary authority”^58^ to the Supreme Court for designing rules and amendments, Congress can amend the Rules directly through legislation.^59^ Thus, Congress could pass legislation that bans the use of plea bargaining in federal courts and incorporate this into the Federal Rules of Criminal Procedure without waiting for the Supreme Court to propose these changes.

While certain rules apply to state and federal courts, others like Rule 11 only apply to federal prosecutions.^60^ As of writing, approximately 11% of all people in confinement in the United States are currently incarcerated in federal prisons,^61^ with the number of prosecutions increasing substantially in recent years.^62^ Though most people in federal prisons have a sentence of less than one year,^63^ federal sentencing penalties are longer than those for similar state crimes.^64^ Additionally, the Federal Rules of Criminal Procedure are often used as a template by state criminal courts to model their own criminal procedures. State and federal legislators participate in policy diffusion, adopting policies from other governments that they believe would work well for their own.^65^ If the federal government were to ban plea bargaining, it is likely that many states would follow suit. Therefore, targeting federal procedures may have a small immediate effect but could lead to broader systemic change in due time.

The legislative process is often faster than a court proceeding, but it is still inhibited by many procedural hurdles, resource scarcity, and, more importantly, by politics and partisanship. Proponents of eliminating plea bargains will need to convince legislators from across the political spectrum to support legislation that would fundamentally alter the carceral process. If the issue becomes partisan, especially if it is taken up by Democrats, the legislation will be further inhibited by the current practice of obstructionism in Congress.^66^ Therefore, advocates would have to find a policy window in which to successfully propose and pass this legislation.^67^

### State or County Interventions

#### Legislation

Like federal legislation, state legislation would not fully eradicate plea bargaining on its own but could make a significant difference in the amount of plea bargains occurring around the country. More than one million people are currently serving sentences in state prisons compared to 200,000 in federal prisons.^68^ State courts are the primary site of prosecutions and thus a significant site for intervention.

States not only learn from the federal government but from each other. Policy diffusion often occurs between states, especially those that share geographic or ideological similarities, and because state legislation tends to move more quickly at the state level, momentum can build, leading to massive change in a short period of time. Additionally, states are sites for policy experimentation and can adopt different policies to suit their unique needs.^69^ These different policies toward similar goals can allow real-time analysis of efficacy and feasibility, whereas a universal approach may have unexpected consequences or hurdles and would be more challenging to modify or overturn for the same reasons that make it challenging to adopt in the first place.^70^

Additionally, many states do not need to rely on legislators alone to change law. California banned plea bargaining in 1982 through a ballot initiative,^71^ a process which can allow for legislation that is preferred by the populace even if the current legislature is opposed to a proposed policy. Ballot initiatives similarly lead to policy diffusion, either because advocates in other states see their effectiveness or because legislators find that their constituents with similar politics may be more supportive than initially presumed.^72^

#### Prosecutorial Interventions

Plea bargains also need not be banned through legislation, though legislation is much more stable and consistent. In 1975, Alaska’s Attorney General, Avrum Gross, banned plea bargains throughout the state;^73^ though the practice resumed in 1985, it was again banned in 2013 through the same mechanism, and remains in place to date.^74^ District Attorneys have also elected to end plea bargaining practices for cases in their jurisdiction.^75^ Attorneys General and District Attorneys are often elected positions,^76^ which grants some constituent power to demand changes to prosecution practices in order to gain and maintain power. Some District Attorneys have run on platforms of “progressive prosecution” in recent years specifically because of constituent demand.^77^

Prosecutorial interventions can be immediate, and they can require a lower threshold of advocacy to enact. Instead of getting enough signatures for a ballot initiative or reaching enough votes in the legislature, a District Attorney or Attorney General could unilaterally halt plea negotiations. However, a major downside is that prosecutorial decisions have no enforcement mechanism as demonstrated by Alaska’s plea bans, where the practice never truly stopped.^78^ Additionally, once the acting District Attorney or Attorney General retires or is voted out, any decisions they made can be easily overturned. Therefore, this pathway, while incredibly feasible, is likely not as effective as others considered in this paper.

## Other Considerations

This paper argues that plea bargains are a primary driver of lengthy incarceration and its harmful effects. Eliminating or severely restricting plea bargaining is likely to provide more opportunities for fair trials and ensure fewer individuals are incarcerated for crimes they did not commit or agreeing to sentence lengths without a judge’s consideration of mitigating information. However, other mechanisms embedded in the carceral system may inhibit the efficacy of any interventions tackling plea bargaining.

### Mandatory Minima

Plea bargains are only as effective as the bargaining power granted to prosecutors. Prosecutors must balance offering attractive concessions for defendants with the State’s interest in enforcing the law, but they are granted significant discretion before a case ever enters the courtroom. Prosecutors ultimately decide which charges will be brought before the court for trial, and if they can demonstrate that they have a factual basis for the plea, they can leverage this power in all negotiations.^79^ In states with mandatory minima,^80^ a person who has been charged with several crimes may be facing decades of prison time, especially if the sentences are not served concurrently.

Mandatory minima work in concert with plea bargaining. Prosecutors rely on the threat of a lengthy sentence to increase their bargaining power. Mandatory minima for certain charges legitimize these prospective calculations. Similarly, “three-strike” laws provide tangible threats to defendants who have prior convictions, as they may be facing disproportionate sentences due to past involvement in the carceral system.^81^ If plea bargains are eliminated but mandatory minimum sentencing is still practiced or enforced, individuals who may have been able to negotiate for certain charges not to be brought forth, lowering the maximum sentence they could face if found guilty, could now be charged with and have to defend against more or higher penalty charges. Should they be unsuccessful in their case, they could end up serving more time than they would have if they had negotiated for certain charges to be dropped or adjusted to a lower penalty.

Even if plea bargains were not eliminated entirely, eliminating mandatory minima could provide similar benefits to defendants as the prosecutor would have less certainty about how many years the defendant may be facing and therefore less leverage. Additionally, for those who pursued trial and were found guilty, their sentence can be determined by the unique circumstances of their case which, for many, would lead to more lenient sentences. In either case, eliminating mandatory minima must be part of the broader effort to address sentence lengths and the public health effects of incarceration.

### Cash Bail

One of the few benefits of plea bargains can be a guaranteed release date. Nearly 500,000 people are currently in pre-trial detention, experiencing virtually the same conditions as they would in prison without having been convicted yet of any crime.^82^ For these individuals, most of whom were simply unable to pay bail,^83^ the idea of bargaining for a shorter sentence rather than chancing an extended period of incarceration and continued punishment can be incredibly enticing. This is especially true if it means they are no longer in “pre-trial” and can just begin serving whatever sentence is ultimately determined.

Advocates have long argued that bail reform is necessary to minimize the harmful effects of incarceration. Bail is often presumed necessary to ensure that only the most dangerous remain incarcerated while awaiting trial; however, in practice, only the poorest remain incarcerated. In a system without plea bargains, where every case would have to go to trial, those who are held in pre-trial detention would necessarily be held longer.^84^ The burdens would be disproportionately felt by vulnerable populations, especially people of color, as Black and brown people are far more likely to be held in pre-trial detention, required to pay bail, and charged higher bail amounts than their white counterparts.^85^ Since 1983, pretrial detention has accounted for 63% of jail population growth. Thus, any policies that attempt to limit incarceration periods must incorporate options that address pre-trial detention rates, and bail reform is a relatively feasible and effective way to do so.^86^

### Overcriminalization

The carceral system serves to punish those who have broken the law. However, what is deemed criminal behavior and effective punishment changes based on sociological, economic, cultural, and political factors.^87^ Most cases that result in plea deals are low-level offenses, many of which criminalize behaviors that many believe should not be addressed through any punitive means. For example, despite all we have learned about addiction, the vast majority of crimes for which people are incarcerated are drug related.^88^ Many other people are incarcerated due to the criminalization of acts of survival, such as panhandling and sleeping outside — behaviors that merely are a result of homelessness.^89^

As long as the jail population remains constant, the system will remain overwhelmed, and plea deals will arguably remain required to maintain some amount of expediency. Decriminalization of all substances, sex work, and behaviors related to homelessness, as well as investment in diversion programs and social programs to address homelessness, hunger, healthcare access, would all but eliminate the need for plea bargaining at the practical level.^90^ These investments not only address the majority of “low level” offenses but may address other entry points to the carceral system by addressing the root causes of violence and incarceration.^91^

## Discussion

Within the current available pathways for intervention, opponents of plea bargaining would have the most success working locally through state legislation to begin changing the judicial system. States are more politically unified than federal governments, and state legislation can be enacted and adopted more quickly than federal legislation or judicial challenges. State legislative efforts also offer opportunities for policy diffusion among neighboring states, states with similar political goals, and states with similar resources to experiment with new policy ideas.^92^ Finally, codifying a ban in state legislation would ensure longevity and accountability in a way that prosecutorial flexibility would not.

There are still debates about whether bans on plea bargains are at all effective in addressing incarceration rates. Two themes can be found in these arguments. First, there are different interpretations of what a ban on plea bargaining would mean. This can be addressed most effectively through a period of policy experimentation by different states or localities to determine the best way to enact and enforce a plea-bargaining ban.

Second and more important, the concern about judicial system gridlock presumes that the number of cases the judicial system currently processes would remain consistent. This can be addressed in a variety of ways. As noted earlier, a ban on plea bargains can lead to prosecutors being more strict when screening cases brought to them by the police, making sure that there is sufficient evidence to pursue charges before filing any cases. Creating alternatives to incarceration through decriminalization and community investment would also minimize the number of individuals engaging with the carceral system in any way.

This paper focuses on one facet of a multi-pronged system designed to disappear community members and justify abuses of human rights. Scholars, activists, and public health leaders argue that the carceral system cannot meaningfully address behaviors deemed criminal and that punitive systems of any kind are not effective, and that efforts at prison reform must not reaffirm the legitimacy of the carceral system as it exists today.^93^ We need to approach all work in addressing the carceral system with this philosophy in mind. Eliminating plea bargaining is merely one step toward that goal. So long as prisons stand, it is imperative that we minimize the number of individuals entering them, the length of their stay, and the collateral consequences they experience upon release.

No matter how they are justified, plea bargains cause people to be incarcerated who may otherwise not be because of the inherent power imbalance between prosecutors and defendants. No amount of reform can truly eliminate that power imbalance. Therefore, it is imperative that prosecutors bear the burden of bringing all cases to trial to ensure that only those with the strongest evidence are moved forward and that all defendants have their constitutional rights protected.

To infringe on fundamental rights, the state must have a compelling interest, and the infringement must be the least restrictive to achieve that goal.^94^ Funneling people through the carceral system to avoid addressing the structural flaws of said system is not a compelling state interest. A system that relies nearly exclusively on individuals rescinding their constitutional rights to function is a fundamentally unjust system.

## Note

Riley Smith is the winner of the 4th annual ASLME Health Law and Anti-Racism Graduate Student Writing Competition.
